# Intergroup Reconciliation between Flemings and Walloons: The Predictive Value of Cognitive Style, Authoritarian Ideology, and Intergroup Emotions

**DOI:** 10.5334/pb.333

**Published:** 2017-11-21

**Authors:** Jasper Van Assche, Dries Bostyn, Jonas De keersmaecker, Benoit Dardenne, Michel Hansenne

**Affiliations:** 1 Department of Developmental, Personality and Social Psychology, Ghent University, BE; 2 Department of Psychology: Cognition and Behavior, University of Liege, BE

**Keywords:** reconciliation, cognitive style, authoritarian ideology, intergroup emotions

## Abstract

Testifying to the gap in fundamental research on positive intergroup outcomes, we investigated reconciliation attitudes in a non-violent intergroup context (i.e., the linguistic conflict in Belgium). By incorporating both important predictors of negative outgroup attitudes (i.e., individual differences in rigid cognitive styles and authoritarian ideologies), and important predictors of reconciliation (i.e., intergroup emotions), we aimed to contribute to a more comprehensive theoretical framework for the analysis of intergroup relations. We recruited one Flemish (*N* = 310) and one Walloon (*N* = 365) undergraduate students sample to test the proposed model. Structural equation analyses with maximum likelihood estimation were conducted using the Lavaan package. In both samples, similar patterns were found. More in particular, the need for cognitive closure appeared to be the basic predictor of right-wing attitudes (i.e., right-wing authoritarianism and social dominance orientation) and essentialist thinking, which were then associated with less outgroup empathy and trust, and more outgroup anger. Furthermore, outgroup trust and empathy were positively related to reconciliation. Interestingly, some differences between the Flemish and Walloon sample were found, such as the direct effects of need for closure and social dominance orientation in the first sample, and the non-significant effects of essentialism in the latter sample. Considering the ongoing public and political debate about the linguistic conflict in Belgium, these findings shed a new light on how individual differences relate to specific outgroup emotions, and how these are associated with important intergroup outcomes in the face of intergroup conflict.

Research on prejudice, stereotyping, discrimination and their correlates has been on the forefront of research agendas in the fields of psychology, sociology, and political sciences since the 1950’s (e.g., Adorno, Frenkel-Brunswik, Levinson, & Sanford, 1950; Allport, 1954). However, this scholarly focus on mere negative outcomes does not provide an answer to the question how *positive* intergroup relations develop (Tropp & Mallett, 2011). Recent accounts (e.g., Nagda, Tropp, & Paluck, 2006; Pittinsky, Rosenthal, & Montoya, 2011) have expanded this narrow focus on intergroup bias and conflict towards an inclusion of positive outgroup attitudes, tolerance, forgiveness, and reconciliation. Reconciliation, defined as the healing of impaired intergroup relations after a period of conflict (Bar-Tal, 2000; 2013), is considered a crucial aspect and even a necessary condition in the challenge of overcoming past troubles and achieving positive intergroup relations (Bar-Tal, 2000).

Reconciliation stems from the Latin verb ‘reconciliare’, roughly translated as ‘making friendly again’, and consists of several components. First, reconciliation refers to a political process and a conflict resolution approach through which the parties in conflict establish a new situation of societal peace, shared goals, and ‘a fusion of horizons’ (Fisher, 1997; Schaap, 2004). Second, fundamental to the reconciliation process is the restoration and rebuilding of relationships between the conflicting groups (Bar-Tal, 2013). To date, the latter approach has only received scant attention in social psychological research. Testifying to this gap in fundamental research on positive intergroup outcomes, we investigated reconciliation attitudes in a non-violent intergroup context (i.e., the linguistic conflict in Belgium). By incorporating both a) important correlates of negative outgroup attitudes (i.e., individual differences in rigid cognitive styles and conservative ideologies), and b) important predictors of reconciliation (i.e., intergroup emotions), we aimed to contribute to a more elaborate and comprehensive theoretical framework for the analysis of intergroup relations.

## Cognitive Style and Authoritarian Ideology

### Need for closure as ‘prejudiced personality’

Contemporary psychological research has accumulated and integrated evidence from two individual difference approaches to explain prejudice and negative outgroup attitudes: a) basic, motivated cognitive styles (i.e., need for closure and essentialist social categorization) and b) conservative, authoritarian ideologies (i.e., right-wing authoritarianism and social dominance orientation). These individual differences underlie prejudice in its different forms, including ethnic prejudice and racism (e.g. Van Hiel, Pandelaere, & Duriez, 2004), sexism (e.g., Roets, Van Hiel, & Dhont, 2012), ageism (e.g., Van Assche, Roets, De keersmaecker, & Van Hiel, 2017), homophobia (e.g., Haslam, Rothschild, & Ernst, 2000), and transgenderism (Tebbe & Moradi, 2012). Roets and Van Hiel (2011a) proposed the Need for (Cognitive) Closure (NFC) as a basic predictor of negative outgroup attitudes, closely related to Allport’s classic conception of ‘the prejudiced personality’ (1954). NFC has been defined as the desire for ‘*an* answer on a given topic, *any* answer, … compared to confusion and ambiguity’ (Webster & Kruglanski, 1994, p. 1049; italics in original); and this cognitive style consists of two tendencies that guide decision-making. First, individuals high in NFC seek to *seize* easily accessible information (i.e., the urgency tendency). Second, they are inclined to *freeze* upon the obtained answer, even by ignoring or minimalizing contradictory information (i.e., the permanence tendency). Applying these tendencies to outgroup evaluations and prejudice, several studies showed that individuals high in NFC want to satisfy their need for quick, easy, firm, and stable knowledge about the social world by categorizing others based on group membership rather than as individuals (Shah, Kruglanski, & Thompson, 1998), and they are also more likely to ‘close off’ their own social environment and derogate outgroups (Webster & Kruglanski, 1994).

### Essentialist thinking and authoritarian ideology as mediators

Roets and Van Hiel (2011a) proposed a mediation model where motivated cognitive style (i.e., NFC) is positively related to various kinds of prejudice via essentialist thinking and authoritarian ideology. Essentialist thinking is another cognitive process that, by allocating meaningful attributes to individuals merely based on their group membership, leads to prejudice (Medin, 1989). Indeed, social categorization is a natural human tendency which allows people to cope with the complexity within the social world by organizing knowledge into categories (Allport, 1954). Individuals high in essentialism (ESS) tend to believe that a social category has meaningful, defining attributes that are shared by all members within this category (e.g., Roets & Van Hiel, 2011b). Such *belief in essence* (Allport, 1954, p. 174; italics in original) expresses the common identity of all members within a social group, thus allowing fast and steady inferences about them and satisfying the need for quick and stable answers about social outgroups. Indeed, previous studies obtained strong positive correlations between NFC on the one hand, and homogeneous perceptions of social groups (Dijksterhuis, van Knippenberg, Kruglanski, & Schaper, 1996), reliance on group membership information when making social judgments (Kruglanski & Mayseless, 1988), and essentialist thinking (Roets & Van Hiel, 2011b) on the other hand. Applied to outgroup evaluations and prejudice, individuals high in essentialist thinking tend to attribute stereotypes of a social category to all members of that group, which enhances several forms of prejudice, including anti-black stereotyping and racism (e.g., Chao, Hong, & Chiu, 2013; Tadmor, Chao, Hong, & Polzer, 2013), and homophobia (e.g., Haslam & Levy, 2006; Haslam et al., 2000).

NFC not only affects the cognitive processes in social judgment, it also shapes ideological beliefs. According to Jost, Glaser, Kruglanski, and Sulloway (2003), people adopt ideological belief systems (in part) because they satisfy their deeper psychological needs and motives. Right-wing ideological beliefs, or conservative social beliefs about the ideal arrangement of society, thus stem from a basic cognitive intolerance and way of dealing with cognitive ambiguity (Roets, Kruglanski, Kossowska, Pierro, & Hong, 2015). Plenty of studies have demonstrated strong positive associations between NFC and the dimensions of right-wing attitudes (e.g., Dhont, Roets, & Van Hiel, 2013; Kossowska & Van Hiel, 2003; Onraet, Van Hiel, Roets, & Cornelis, 2011). Right-wing attitudes often fall apart into two related^1^ dimensions, which are usually labeled right-wing authoritarianism (RWA) and social dominance orientation (SDO; see Duckitt, 2001; Duckitt & Sibley, 2007; 2009; 2010). RWA (i.e., a generalized social belief encompassing the adherence to traditional social norms, aggression towards those deviating from these norms, and the craving for authorities that impose discipline; Adorno et al., 1950; Altemeyer, 1981) is an important social attitude in the prediction of prejudice. According to Duckitt (2001; see also, Van Hiel, Cornelis, & Roets, 2007), RWA can be considered the social-cultural dimension of right-wing attitudes. People high in RWA generally see the world as a dangerous place, and are therefore motivated to preserve social cohesion, conformity, and security, and to react negatively towards outgroups (see Duckitt & Sibley, 2010; Van Assche, Roets, Dhont, & Van Hiel, 2014). Furthermore, several studies have shown that the relations between NFC and subtle racism (Onraet et al., 2011; Roets & Van Hiel, 2006; Van Hiel et al., 2004), implicit racism (Cunningham, Nezlek, & Banaji, 2004) or sexism (Roets et al., 2012) are largely explained by RWA, as individuals high in NFC satisfy their need for fast and steady knowledge about the social world by adhering to this ideological belief system.

Finally, SDO, denoting the preference for hierarchy and inequality between social groups (Pratto, Sidanius, Stallworth, & Malle, 1994) can be considered as the economic dimension of right-wing attitudes. People high in SDO are likely to perceive the world as a competitive jungle, and are therefore inclined to maintain the status quo within the social hierarchy, and to react biased towards outgroups (e.g., Duckitt & Sibley, 2007; 2009; 2010). Adhering to this ideological belief system can thus also satisfy NFC, since the preservation of the societal status-quo and the placement of in- and outgroups on the ‘social-hierarchical ladder’ offers epistemic security (Roets et al., 2011a; 2015). Noteworthy, models testing NFC effects through SDO on prejudice generally showed weaker and less pronounced mediating effects (Roets et al., 2011a; 2015). However, it can be concluded that both dimensions of conservative, authoritarian ideology are strongly linked to both NFC and to negative outgroup attitudes.

## Intergroup Emotions and Reconciliation

Whereas the aforementioned individual difference variables have found to be positively related to various types of negative outgroup attitudes, it remains relatively unknown how strongly they are (negatively) related to positive intergroup outcomes, such as reconciliation. Some studies already linked individual differences with intergroup emotions (e.g., Bäckström & Björklund, 2007; Dhont & Van Hiel, 2011; Van Assche, Roets, Dhont, & Van Hiel, 2016), which are among the most potent predictors of reconciliation attitudes (e.g. Cehajic, Brown, & Castano, 2008; González, Manzi, & Noor, 2011; Tam et al., 2007). Therefore, we suggest that intergroup emotions play a vital role in the relation between individual differences and reconciliation.

From a componential perspective (Fontaine & Scherer, 2013; Scherer, 2005), the construct of emotion can be divided into six components (i.e., appraisal of events, subjective experiences, emotion regulation, action tendencies, motor expressions, and psycho-physiological changes). Although there is some intergroup research focusing on cognitive appraisal and action tendencies (e.g., Mackie, Devos, & Smith, 2000), intergroup emotions are usually examined as subjective feelings towards outgroups. This subjective component is experienced by individuals when they identify with a social group and regulates intergroup attitudes and behavior (Smith, Seger, & Mackie, 2007). Positive outgroup emotions have been associated with more reconciliation and forgiveness attitudes. For instance, the interrelated positive outgroup emotions of trust and empathy predict reconciliation attitudes in several fractured (post-) conflict areas, such as Bosnia and Herzegovina (e.g., Cehajic et al., 2008), Chile (e.g., Noor, Brown, González, Manzi, & Lewis, 2008), Northern Ireland (e.g., Tam et al., 2007), Israel (e.g., Nadler & Liviatan, 2006), and South Africa (Swart, Hewstone, Christ, & Voci, 2011). Alternatively, negative intergroup emotions, such as anger, have been related to less reconciliation (e.g., Halperin, 2011; Iyer, Schmader, & Lickel, 2007; Tam et al., 2007). In this study, we investigated the role of three intergroup emotions: outgroup trust, empathy, and anger (see González et al., 2011).

### Outgroup trust

Alarcón-Henríquez and colleagues (2010) examined the pivotal role of outgroup trust and suggested that trust is a necessary condition for creating positive attitudes and promoting reconciliation. By restoring trust, the two conflicting groups create a new dynamic in their relations, without staying fixated on the past (see also, Nadler & Liviatan, 2006). Trust knits society together, and therefore, trust building is essential in de-escalating conflict and facilitates social relations and future cooperation among groups in past conflict settings (Tam, Hewstone, Kenworthy, & Cairns, 2009). In other words, trust is often considered the basic ‘thrust’ of reconciliation (Bar-Tal, Halperin, & De Rivera, 2007), and individual differences in (outgroup) trust might therefore be strongly positively related to individual differences in reconciliation attitudes. Moreover, several studies indicated that right-wing ideological attitudes are negatively related to outgroup trust (see e.g., Dhont & Van Hiel, 2011; Van Assche et al., 2016). Therefore, we expected that rigid cognitive styles (i.e., NFC and ESS) and right-wing attitudes (i.e., RWA and SDO) are associated with reconciliation through outgroup trust.

### Outgroup empathy

In the same vein, research on outgroup empathy generally indicated that it serves as a potent predictor of positive outgroup attitudes (Batson et al., 1997; Finlay & Stephan, 2000; Swart et al., 2011). In intergroup relations literature, two components of the multifaceted construct of empathy (Davis, 1983) are considered to be essential in how one views social outgroups. First, empathizing with members of the other group provides appreciation of their emotional state and feelings (i.e., empathic concern). Second, insight into the viewpoint (i.e., perspective taking) of this other group serves as a crucial step towards mutual understanding, forgiveness and, eventually, reconciliation (Pedersen, Beven, Walker & Griffiths, 2004). Noor and colleagues (2008) revealed that participants high on outgroup empathy had increased scores on reconciliation indices. Cehajic and colleagues (2008) further showed that both trust and empathy are strongly positively related to reconciliation and forgiveness (see also, Nadler & Liviatan, 2006). Moreover, authoritarian attitudes have already been negatively related to outgroup empathy (Bäckström & Björklund, 2007; Nicol & Rounding, 2013). Hence, we propose empathy as our second outgroup emotion bridging individual dispositions and reconciliation attitudes.

### Outgroup anger

Whereas previous research highlights the vital role of trust and empathy as positive affective predictors of reconciliation, outgroup anger is often considered an enemy of positive intergroup relations (see e.g., González et al., 2011; Halperin, 2011). Years of conflict leave deep scars of anger towards the other group, often accommodated with long-lasting feelings of grudge, resentment, hate, and a sense for revenge. Indeed, outgroup anger seems to be an inherent component of intergroup conflict, and studies on the invasion in Iraq (Iyer et al., 2007), the ideological conflict in post-Pinochet Chile (Manzi & González, 2007), and the religious conflict in Northern Ireland (Tam et al., 2007) all demonstrated that people are not ready to forgive unless their feelings of anger towards the opposing group have diminished. Tam and colleagues (2007) especially pointed to the positive relation between anger on the one hand and refusal to negotiate, compromise, and reconcile on the other. Likewise, Halperin (2011) indicated that anger directed towards an adversary serves as a barrier to reconciliation and support for peaceful conflict resolution. Finally, Manzi and González (2007) found clear and consistent negative associations between outgroup anger and reconciliation in an offender as well as a victim sample. It is thus reasonable to assume that such anger will inhibit the willingness to reconcile. Whereas one study (Heaven & Bucci, 2001) showed no association between RWA/SDO and general anger (measured as a personality trait), no studies investigated the link between such individual dispositions and specific outgroup anger.

## The Present Study

**Figure 1:**
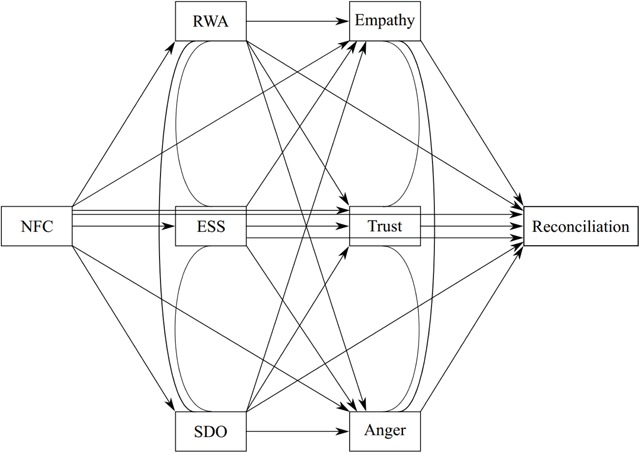
Conceptual Model.

In order to examine reconciliation attitudes, it thus seems vital to include a) individual difference variables as well as b) intergroup emotions. In line with Roets and Van Hiel (2011a), we propose NFC to exert its influence on outgroup emotions via RWA, SDO, and ESS. Furthermore, we allow these individual differences to relate to all intergroup emotions. As intergroup emotions are more proximal to reconciliation attitudes, we propose that the rigid cognitive styles and right-wing social attitudes are related to the experience of less trust, less empathy, and more anger towards the outgroup, which, in turn, are associated with more reconciliation. This leads to a conceptual model with one basic predictor (i.e., NFC), three individual dispositions (i.e., RWA, SDO, and ESS), followed by three outgroup emotions (i.e., trust, empathy, and anger) that eventually relate to one outcome (i.e., reconciliation; see Figure 1).

We investigated this model with an explorative structural equation modeling approach in Belgium. Belgium has a long-standing history of tensions and non-violent conflict between the two main linguistic communities, the Flemish speakers (Flemings) and the French speakers (Walloons^2^; Klein, Licata, Van der Linden, Mercy, & Luminet, 2012). Though not violent, recurrent linguistic and territorial conflicts have polarized Flemings and Walloons, and have profoundly shaped the nation ever since its establishment in 1831 (Covell, 1981). When Belgium was founded, French was the only official language. The Dutch-speaking felt repressed, which resulted in a Flemish movement that advocated for more recognition (Vos, 2002). Due to these efforts, Dutch became an official language in 1898. In 1962, the Belgian parliament approved a law that fixed a permanent linguistic border in Belgium. Afterwards, disputes arose regarding French-speaking boroughs surrounding Brussels and the expansion of the French language around Belgium’s capital, Brussels. Brussels is an autonomous bilingual region within the territory of Flanders with a large share of French-speakers, and its citizens do not typically consider themselves Flemish or Walloon. Therefore, people from Brussels were not allowed to partake in the current study.

In short, Belgium seems a most interesting country to investigate reconciliation attitudes. Nonetheless, reconciliation attitudes have rarely been examined in this non-violent conflict setting (for a notable exception, see Alarcón-Henríquez et al., 2010). Because of the exploratory nature of this study, we tested the full model in a Flemish and a Walloon sample, as such aiming to investigate the generalizability of the obtained relationships as well as to identify possible group-specific effects for one or the other linguistic community.

## Method

### Participants

Three hundred and ten Dutch-speaking undergraduate psychology students without migration background^3^ from a Flemish university (81.6% women, *M*_age_ = 19.77 years, *SD*_age_ = 3.94) participated in return for partial course credit. Furthermore, 365 French-speaking undergraduate psychology students without migration background from a Walloon university (77,5% women, *M*_age_ = 20.65 years, *SD*_age_ = 4.28) partook in the study. All participants completed the full online questionnaire.

### Measures

All measures were rated on 7-point Likert scales anchored by 1 (strongly disagree) and 7 (strongly agree), except for the NFC scale which is traditionally rated on a 6-point Likert scale. To obtain reliable translations, two bilinguals independently back-translated each other’s translation of each measure from Flemish to French or vice versa. Afterwards, all authors compared the original and back-translated version to inspect their equivalence in order to arrive at consensual agreement on the final wording (for a similar procedure, see e.g., Chen, Van Assche, Vansteenkiste, Soenens, & Beyers, 2015).

#### Need for closure

Participants completed the 15-item version (Roets & Van Hiel, 2011c) of the revised NFC-scale (Roets & Van Hiel, 2007; original version by Webster & Kruglanski, 1994). An example item is: *‘I don’t like situations that are uncertain’*.

#### Right-wing authoritarianism

Participants responded to an 11-item version of Altemeyer’s (1981) RWA-scale (see e.g., Roets & Van Hiel, 2006). An example item is: *‘Obedience and respect for authority are the most important virtues children should learn’*.

#### Essentialism

A 12-item version of the Essentialist Entitativity scale was administered (see also, Roets & Van Hiel, 2011b). An example item is: *‘Members of a certain group are usually very similar’*.

#### Social dominance orientation

To measure SDO, Pratto, Sidanius, Stallworth and Malle’s (1994) 14-item scale was administered. An example item is: *‘Some groups of people are simply inferior to other groups’*.

#### Outgroup empathy

Empathy was measured with two items adapted from Pedersen and colleagues (2004). These items read *‘I often feel sympathy for the Walloon [Flemish] Community’*, and *‘I am not able to understand the Walloon [Flemish] point of view during community debates’* (reverse coded).

#### Outgroup trust

Outgroup trust was measured with two items adapted from Cehajic and colleagues (2008): *‘Despite the recent events that occurred during the linguistic conflict, I am hopeful and trust the Walloons [Flemings]’*, and *‘Trust is an important step towards the reconciliation of our two linguistic communities’*.

#### Outgroup anger

Outgroup anger was measured with two items, adapted from Brown, González, Zagefka, Manzi and Čehajić (2008). These items were *‘Thinking about how Walloons [Flemings] have treated Flemings [Walloons] makes me feel angry and irritated’*, and *‘I feel humiliated and indignant when I think about how the Walloons [Flemings] treated Flemings [Walloons]’*.

#### Reconciliation

To measure reconciliation, participants responded to an 11-item scale. These items were based on former scales (see Tam et al. 2007; Philpot & Hornsey, 2008) and were adapted to the specific context of the linguistic conflict in Belgium. We aimed to select specific items that represent reconciliation beyond mere forgiving, as such tapping into the emotional (e.g., *‘It is important that we let go our negative feelings towards Walloons [Flemings]’*), cognitive (e.g., *‘I think that cooperation with the Walloons [Flemings] is necessary and of mutual interest’*), and behavioral component of reconciliation (e.g., *‘I would want Flemings [Walloons] to avoid Walloons [Flemings] as much as possible’*; reverse coded).

## Results

### Correlations and Descriptive Statistics

Means, standard deviations and reliability statistics for each measure are presented in Table 1; as are the correlations between each of the measures. Crucially, in both samples, all measures displayed acceptable reliability (all *α*s > .73). Though all measures were significantly correlated with one another in the Flemish sample, this appeared not to be the case in the Walloon sample. Especially the correlational pattern of NFC differed between both samples, with NFC not being significantly correlated with SDO, outgroup empathy, and reconciliation in the Walloon sample. Furthermore, although SDO and outgroup anger were significantly interrelated in the Flemish sample, they were unrelated in Walloon sample. Overall, the pattern of associations was largely similar in both samples, though this correlational analysis does already suggest some intriguing differences between both samples.

**Table 1:** Variable Means, Standard Deviations (SD), Cronbach’s αs and Correlations.

	Mean *(SD)*	α^a^	1	2	3	4	5	6	7

FLEMISH SAMPLE									

1. NFC	3.75 *(0.63)*	.82							
2. RWA	3.32 *(0.91)*	.82	.43***						
3. Essentialism	3.70 *(0.67)*	.75	.15*	.28***					
4. SDO	2.91 *(0.95)*	.88	.14*	.46***	.33***				
5. Empathy	4.62 *(1.04)*	.30***	–.23***	–.32***	–.24***	–.25***			
6. Trust	5.01 *(0.88)*	.47***	–.17**	–.23***	–.24***	–.28***	.53***		
7. Anger	2.75 *(1.32)*	.52***	.26***	.28***	.20***	.18**	–.31***	–.32***	
8. Reconciliation	5.45 *(0.73)*	.83	–.14*	–.27***	–.28***	–.35***	.52***	.70***	–.33***
**WALLOON SAMPLE**									

1. NFC	3.54 *(0.64)*	.79							
2. RWA	3.51 *(0.78)*	.73	.35***						
3. Essentialism	3.82 *(0.67)*	.75	.16**	.19***					
4. SDO	2.83 *(0.86)*	.83	.08	.37***	.18***				
5. Empathy	4.21 *(1.01)*	.23***	–.06	–.22***	–.14**	–.16**			
6. Trust	4.68 *(1.05)*	.48***	–.10^†^	–.24***	–.13*	–.25***	.57***		
7. Anger	3.76 *(1.28)*	.66***	.17**	.27***	.14**	.04	–.37***	–.33***	
8. Reconciliation	5.13 *(0.70)*	.79	–.08	–.27***	–.18**	–.44***	.48***	.54***	–.27***

*Notes*: ^†^: *p* < .10; *: *p* < .05; **: *p* < .01; ***: *p* < .001. ^a^: for two-item measures, the inter-item correlation is provided instead of α.

### Structural Equation Model

To test the model, we adopted an explorative structural equation modeling approach with the Lavaan package (version0.5-20; Rosseel, 2012) in R (version 3.2.3; R Core Team, 2015). All measures, except for the outgroup emotions, were introduced into the model as latent variables, using the individual items as indicators. We did not include the outgroup emotions into the model as latent variables as these were measured with only two items, and three items are a minimum for measuring latent constructs. Before fitting the model, we tested for multivariate normality through a Mardia’s test with the MVN package in R (Mardia, 1970; Korkmaz, Goksuluk & Zararsiz, 2014). All three sub-tests rejected the null hypothesis of multivariate normality (all *p* < .001). While this does not necessarily mean that parameter estimates would be biased, it does suggest that a standard maximum likelihood approach would underestimate the standard errors associated with each effect. As such, we used a restricted maximum likelihood approach with robust standard errors and a Satorra-Bentler correction for all reported chi-square goodness of fit tests.

Prior to assessing the structural part of the model, we tested for measurement invariance to ensure that all latent constructs were measured similarly across samples. To do so, we fitted a sequence of increasingly more restricted models; comparing each of these for goodness of fit (Kline, 2015). More specifically, measurement invariance was tested by analysis of four models: a) a model without equality constraints to test for configural invariance; b) a model with equality of the factor loadings to test for weak invariance; c) a model with equality of both factor loadings and intercepts to test for strong invariance; and d) a model with equality of factor loadings, intercepts and latent means to test for strict invariance. Whereas the first two forms of invariance are quite essential for our purposes, as they are necessary to demonstrate that we measured the same constructs across both samples, there is no need to establish strong or strict invariance in the current study. Strict invariance, for instance, would imply that Walloons and Flemings demonstrate similar mean levels for all measured variables. While this would undoubtedly be an interesting finding, there is no a priori reason to assume this to be the case. In fact, we would consider this to be somewhat unlikely given the known ideological differences between both linguistic groups (see Billiet, Maddens, & Frognier, 2006).

Ideally, goodness of fit for each model would be demonstrated through the use of chi-square tests. However, for studies with large samples, complex models and non-normal data, as is the case in the current study, even slight deviations between the observed and model implied covariance matrix will yield chi-square tests that are highly significant, over-rejecting models that nevertheless fit the data quite well (Kline, 2015). As such, many researchers recommend using fit indices instead to help determine if a model fits; both when determining the individual fit of a model and in the context of sequential testing for measurement invariance. In the context of overall model fit, Hu and Bentler (1999) suggest that models with an SRMR ≤ .08 in addition to an RMSEA of ≤ .06 fit very well^4^. In the context of testing for measurement invariance, Chen (2007) suggests that changes of bigger than .030 to the SRMR and bigger than .015 to the RMSEA would designate a break in invariance. Table 2 displays the relevant fit indices for the series of models used to test for measurement invariance. Interestingly, if we follow the recommended cut-offs for testing measurement invariance, it appears that we can assume strict invariance between both samples. However, it is also quite obvious that goodness of fit does decrease quite a bit when going from weak to strong invariance and that the strong invariance model no longer adequately fits the data as the SRMR >.09 and RMSEA = .07. Because of this, and because of the explorative nature of the current study, we preferred a more conservative approach assuming only metric invariance in the subsequent analyses.

**Table 2:** Measurement Invariance: Multi-group CFA fit indices.

Model Description	*χ^2^*	Scaling Factor	*df*	*Δχ^2^*	*Δdf*	*p*	SRMR	RMSEA	ΔSRMR	ΔRMSE

Model 1 (configural invariance)	7622.26	1.13	3760			<.001	.084	.057		
Model 2 (metric invariance)	7821.02	1.13	3818	198.76	58	<.001	.089	.057	.005	<.001
Model 3 (scalar invariance)	9689.86	1.05	3876	1868.84	58	<.001	.095	.069	.006	.012
Model 4 (strict factorial invariance)	9766.97	1.05	3881	77.11	5	<.001	.096	.069	.001	<.001

*Note*: *χ^2^* refers to the Satorra-Bentler scaled chi-square statistic. SRMR: Standardized Root Mean square Residual. RMSEA: Root Mean Square of Error of Approximation.

**Figure 2:**
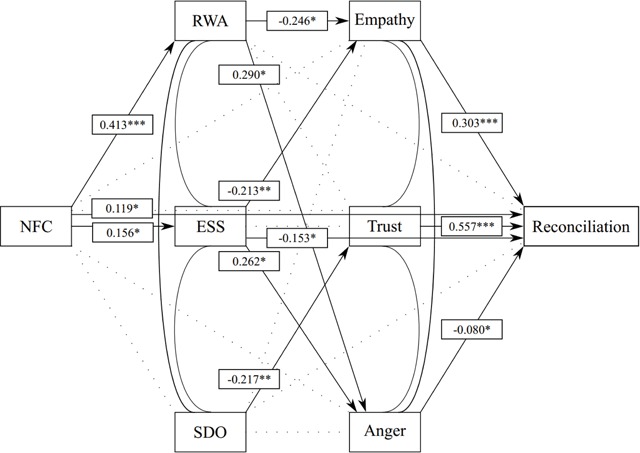
Flemish Model. *Note*: Coefficients denote standardized parameter estimates.

**Figure 3:**
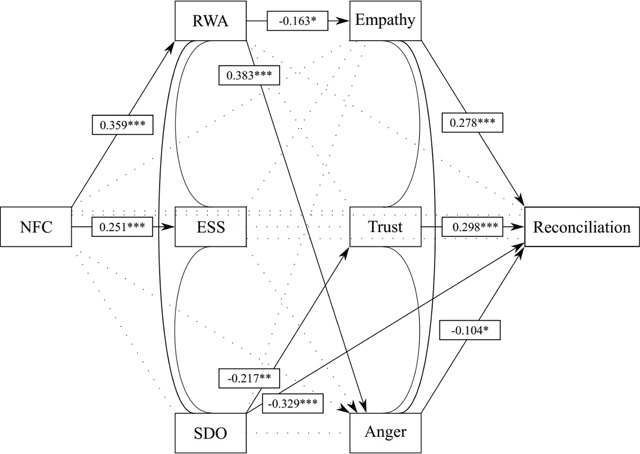
Walloon Model. *Note*: Coefficients denote standardized parameter estimates.

We then progressed by fitting the full model displayed in Figure 1 for both samples, fixing the latent factor loadings to be equal across groups, but freely estimating intercepts, and covariances. The model fitted the data fairly well: *χ2*(4166) = 8457.68, *p* < .001; *χ2/df* = 2.03; RMSEA = .057, SRMR = .088, though the SRMR is on the higher side of what constitutes an acceptable model fit. Table 3 displays all coefficients of the structural model and their associated *p*-values. For ease of interpretation, these results are also summarized in Figures 2 and 3. Additionally, though mediational analyses are not the focus of the current manuscript, all indirect effects are presented in the supplementary materials.

**Table 3:** SEM: Coefficients of the Structural Model.

Regressions:		Flemish Sample (*N* = 310)				Walloon Sample (*N* = 329)			

From	To	Est.	SE	Std. Est.	*p*	Est.	SE	Std. Est.	*p*

Empathy	Reconciliation	0.086	0.023	0.303	<.001	0.077	0.023	0.278	.001
Trust	Reconciliation	0.158	0.035	0.557	<.001	0.082	0.023	0.298	<.001
Anger	Reconciliation	–0.023	0.011	–0.080	.040	–0.029	0.014	–0.104	.044
RWA	Reconciliation	0.007	0.023	0.022	.775	–0.005	0.025	–0.016	.826
SDO	Reconciliation	–0.023	0.020	–0.070	.265	–0.115	0.034	–0.329	.001
ESS	Reconciliation	–0.069	0.029	–0.153	.017	–0.017	0.023	–0.041	.470
NFC	Reconciliation	0.061	0.030	0.119	.043	–0.008	0.029	–0.017	.773
RWA	Empathy	–0.259	0.101	–0.246	.010	–0.202	0.088	–0.163	.021
SDO	Empathy	–0.055	0.092	–0.048	.552	–0.129	0.091	–0.102	.178
ESS	Empathy	–0.337	0.121	–0.213	.005	–0.128	0.095	–0.086	.157
NFC	Empathy	–0.066	0.128	–0.036	.608	0.051	0.110	0.029	.645
RWA	Trust	–0.028	0.084	–0.027	.737	–0.138	0.101	–0.112	.172
SDO	Trust	–0.247	0.112	–0.217	.002	–0.276	0.095	–0.217	.004
ESS	Trust	–0.214	0.080	–0.136	.057	–0.075	0.113	–0.050	.510
NFC	Trust	–0.144	0.109	–0.080	.178	–0.67	0.130	–0.038	.609
RWA	Anger	0.305	0.130	0.290	.019	0.474	0.127	0.383	<.001
SDO	Anger	–0.122	0.112	–0.107	.275	–0.205	0.116	–0.162	.076
ESS	Anger	0.414	0.160	0.262	.010	0.208	0.144	0.139	.149
NFC	Anger	0.302	0.170	0.167	.076	0.107	0.155	0.062	.491
NFC	RWA	.708	0.140	0.413	<.001	0.502	0.118	0.359	<.001
NFC	SDO	.077	0.106	0.049	.465	0.027	0.087	0.020	.758
NFC	ESS	.178	0.088	0.156	.043	0.291	0.090	0.251	.001
**Covariances:**		**Flemish Sample (*N* = 310)**				**Walloon Sample (*N* = 329)**			

**From**	**To**	**Est.**	**SE**	**Std. Est.**	***p***	**Est.**	**SE**	**Std. Est.**	***P***

Empathy	Trust	0.360	0.051	0.360	<.001	0.477	0.066	0.477	<.001
Empathy	Anger	–0.222	0.074	–0.222	<.001	–0.405	0.078	–0.405	<.001
Trust	Anger	–0.257	0.062	–0.257	<.001	–0.319	0.085	–0.319	<.001
RWA	SDO	0.376	0.064	0.496	<.001	0.199	0.045	0.336	<.001
RWA	ESS	0.194	0.044	0.358	<.001	0.112	0.039	0.229	.004
SDO	ESS	0.211	0.046	0.385	<.001	0.091	0.040	0.179	.023

*Note*: NFC: need for closure; ESS: essentialism; RWA: right-wing authoritarianism; SDO: social dominance orientation.

The models for the Flemish and Walloon sample share some important similarities, though they also diverge on some aspects. As for the similarities, it is most interesting that in both samples NFC was associated with RWA and ESS but not SDO (despite a significant bivariate correlation in the Flemish sample). Furthermore, for both groups, the largest drivers of reconciliation appear to be the outgroup emotions of empathy and trust, with anger playing a much smaller yet still significant role. Additionally, in both samples, empathy was driven primarily by RWA, whereas trust was primarily associated with SDO. We also note three important divergences between the Flemish and the Walloon final model results. First, after controlling for all other measures included in the study, NFC was positively associated with reconciliation in the Flemish sample; an association that was absent in the Walloon sample. Second, SDO had a direct association with reconciliation in the Walloon sample, but not in the Flemish sample. Finally, and perhaps most importantly, essentialist thinking was not associated with reconciliation or any of the three outgroup emotions studied in the Walloon sample, whereas it was significantly related to empathy, anger and reconciliation in the Flemish sample. This might reflect an important difference between both groups.

## Discussion

In this contribution, we focused on some social psychological factors that are thought to play a key role in the process of reconciliation, with a particular emphasis on individual dispositions and intergroup emotions. Building on previous findings, this study’s primary objective was to examine a model bringing together and bridging individual difference theories in ideology and cognitive style on the one hand, and outgroup emotions and reconciliation on the other. We tested our model in a Flemish and Walloon sample, and in line with our expectations, we found very similar patterns in both samples, though the paths tended to be a little more pronounced in the Flemish sample. The results revealed a) positive associations between NFC on the one hand, and essentialist thinking and authoritarian attitudes on the other hand, b) negative associations between authoritarian attitudes and positive outgroup emotions and positive associations between authoritarian attitudes and negative outgroup emotions, and c) positive associations between positive outgroup emotions and reconciliation and negative associations between negative outgroup emotions and reconciliation. As such, the models showed that both Flemings and Walloons high in NFC tended to be higher in RWA and essentialist thinking, and were inclined to be less reconciled towards the other linguistic group, because they experienced less trust, less empathy, and more anger towards this outgroup.

### Similarities between the Flemish and the Walloon model

#### Cognitive style and authoritarian ideology

More specifically, in both models, we found effects of NFC on outgroup emotions via RWA. Indeed, to meet their desire for epistemic security and closure in the social environment, people typically resort to authoritarianism, which represents one of the most powerful, proximal determinants of stereotyping, prejudice, and outgroup attitudes (see also, Roets et al., 2011a; 2015). Notably, though NFC is related to other cognitive styles such as intolerance of ambiguity (Frenkel-Brunswik, 1949), uncertainty orientation (Sorrentino & Short, 1986), and need for cognition (Cacioppo & Petty, 1982), and to personality traits such as conscientiousness and (a lack of) openness to experience (McCrae & Costa, 1985), only NFC yields such strong and unique effects on outgroup attitudes via RWA (see e.g., Onraet et al., 2011; Roets et al., 2015). Interestingly, NFC and SDO were not significantly interrelated in both samples. This is not too surprising because the association of NFC with SDO is not overly strong and a few studies have already noted a similar less pronounced association (see e.g., Roets & Van Hiel, 2011a). In order to reach epistemic security and cognitive closure, compliance with traditional norms and values and submission to authorities thus seems a better strategy as opposed to a hierarchy-driven social-ideological belief system focusing on ingroup dominance and the status quo in the relations between the different linguistic groups.

Moreover, RWA was negatively linked to reconciliation via less outgroup empathy and more outgroup anger, but not via outgroup trust. With regards to trust, this was rather unforeseen, as previous studies using Flemish and Dutch data show that this association is quite robust (Dhont & Van Hiel, 2011; Van Assche et al., 2016). Admittedly, those studies investigated trust towards immigrants as an outgroup, and arguably, individuals high in RWA are likely to perceive immigrants as a greater threat to traditional society as opposed to fellow citizens who have been living in Belgium since its foundation. With regard to empathy, our results are in line with previous findings (Bäckström & Björklund, 2007; Nicol & Rounding, 2013) showing that high authoritarians display less empathic concern and are lower in perspective taking. It should be noted that we replicated these results using a less-than-perfect empathy measure, consisting of two weakly correlated items (i.e., one empathic concern item and one reverse coded perspective taking item). With regards to outgroup anger, the positive association with RWA might indicate that a general state of anger (which is unrelated to RWA, see Heaven & Bucci, 2001) can be conceptually differentiated from specific feelings of anger towards an outgroup (which are positively related to RWA). Furthermore, SDO was negatively linked to reconciliation via less outgroup trust. This is, to our knowledge, the first empirical evidence of a negative relation between SDO and outgroup trust. Future studies could further delineate the specific associations between RWA and empathy and anger, on the one hand, and SDO and trust on the other hand.

#### Intergroup emotions and reconciliation

A last similarity in both models pertains to the positive relations of outgroup trust and empathy with reconciliation, and the small but still significant anger-reconciliation association. There is ample evidence that relates both empathy and trust to more support for intergroup reconciliation (e.g., Cehajic et al., 2008; Nadler & Leviatan, 2006; Tam et al., 2009), which was replicated in these two Belgian samples. However, while some studies found a clear negative link between outgroup anger and reconciliation (Manzi & González, 2007; Tam et al., 2007), our findings indicated that this relation is relatively small when other outgroup emotions (outgroup trust and empathy) were included. In the same vein, Halperin (2011) demonstrated that the effect of outgroup anger was rendered insignificant when outgroup hate was taken into account. Halperin (2011) suggested that whereas anger is a strong emotion, it is also a complicated and ambiguous one. Because anger often is characterized by a high level of arousal, it is usually more strongly associated with an approach orientation and with intentions to take action against the outgroup (see also, Iyer et al., 2007) rather than with mere support for intergroup reconciliation.

Furthermore, it is more likely that positive outgroup emotions are stronger predictors of a positive outcome compared to negative emotions (which are more potent predictors of negative outcomes, see Pittinsky et al., 2011). If we would have considered a negative outcome such as prejudice, outgroup aggression, or confrontation intentions, maybe anger would have shown up to be a better predictor. Finally, the weaker relation could be due to the wording of the second item, which combines two related though distinct feelings (i.e., indignation and humiliation) that might drive outgroup anger. Similarly, the weaker association could be due to the selection of reconciliation items beyond mere forgiveness. Previous studies (Iyer et al., 2007; Manzi & González, 2007; Tam et al., 2007) indicated that people are not ready to forgive unless their anger has diminished. As such, some degree of forgiveness may be required for reconciliation to begin. Notably, though forgiveness and reconciliation seem strongly intertwined (see Noor et al., 2008), there can be forgiveness without reconciliation and reconciliation without forgiveness (Murphy, 2000). For example, it is possible to overcome negative emotions without hoping to, or even wanting to, restore a relationship with the offender. Conversely, a victim may attempt reconciliation even while still strongly feeling anger or resentment. Future studies, using multiple-item scales (that fully capture all components of an emotion and of both forgiveness and reconciliation) could further explore the exact role outgroup anger (and general anger) plays in positive and negative outgroup attitudes as well as positive and negative behavioral intentions towards outgroups.

### Differences between the Flemish and the Walloon model

#### The association between NFC and reconciliation

Importantly, we found some interesting differences which we did not expect originally. One such difference is that after controlling for all other measures included in the study, NFC was positively associated with reconciliation in the Flemish sample, but not in the Walloon sample. A closer look at the standardized coefficient of the structural model in the Flemish sample (see Table 3 and Figure 3) shows, however, that this is a rather small effect. Nonetheless, it might be an intriguing hypothesis that, after cancelling out the aspects of NFC related to right-wing attitudes and outgroup negativity, individuals high in NFC perceive reconciliation as the most efficient to reach epistemic security and cognitive closure. Perhaps, as opposed to Walloons, Flemings high in NFC regard the Belgian linguistic conflict more as nearing solution. As such, reconciling to them offers an easy and firm answer to the complex and ambiguous situation dividing Belgium nowadays.

#### The association between SDO and reconciliation

Another difference between the Flemish and Walloon results is that SDO had a direct negative association with reconciliation in the Walloon sample, and not in the Flemish sample. The remaining direct effect specifies that part of SDO’s impact is not emotionally driven for Walloons. Indeed, though high-SDO Flemings and Walloons both tend to refrain from reconciling because they distrust the other group, Walloons high in SDO (but not Flemings high in SDO) are more likely to *rationally* discard reconciliation. A possible explanation would be that Walloons high in SDO rationally tend to reject reconciliation with Flemings because they feel superior, want to preserve the current hierarchy and inequality between the two linguistic groups, and as such do not feel the need to reconcile (Pratto et al., 1994). Nonetheless, because Wallonia these days is considered to be an economically less flourishing region compared to Flanders (see Rochtus, 2012), it is especially surprising that only Walloons high in SDO adopt system-justifying beliefs that are associated with less willingness to reconcile. Future research is needed to clarify how quite different phenomena such as support of current economic inequality and willingness to reconcile are linked.

#### The role of essentialism

Contrary to the results in the Flemish sample, essentialism was not significantly related to any outgroup emotion in the Walloon sample. Though the standardized coefficients of the structural model in this sample (see Table 3 and Figure 3) revealed that the associations are all in the expected direction, the non-significant links suggest that Walloons’ cognition of Flemings forming a distinct and meaningful outgroup (with defining attributes shared by everyone) has less emotional consequences than it does for Flemings high in essentialism. Perhaps high essentialist Walloons do not to base their outgroup emotions on their cognitions, but rather on their authoritarian beliefs. This is a tentative hypothesis. Consequently, as this was the first empirical study linking essentialist thinking to these three outgroup emotions, future studies could investigate exactly why the ‘belief in essence’ is more strongly related to outgroup trust, empathy and anger (and possibly other outgroup emotions, such as envy and despise, too) in some social groups as opposed to others.

### Theoretical and Practical Implications

Reconciling includes (re)building mutual trust, showing sensitivity for the needs and interests of the other group, and letting go of negative feelings and attitudes (Bar-Tal, 2000; 2013). Our findings suggest that the promotion of positive outgroup emotions like trust and empathy might be a significant point of departure for reconciliation processes to occur. We aspire to propose possible interventions for improving intergroup relations between the two main linguistic groups in Belgium. Such interventions should focus on advertising mutual trust, enhancing concern and appreciation of the others’ feelings, and learning to take the perspective of the other group. Nonetheless, although individual dispositions and emotions are important for reconciliation between groups, it is warranted to take into account the broader, macro-level social context as well (Vollhardt & Twali, 2016).

Historically, there has been economic disparity between the two parties (Klein et al., 2012). Before World War II, Wallonia was much wealthier, but afterwards, Flanders turned this situation around. Nowadays, a very popular Flemish political party (i.e., N-VA) considers Wallonia more as a burden than as an ally (Rochtus, 2012; Van Assche et al., 2017). N-VA calls for more autonomy, while Walloon political parties insist on solidarity. This long history of disputes and the current power differences in terms of social-economic status undoubtedly had its effects on how Flemings and Walloons regard and interact with one another. Notably, our results are based on student samples, so we should be careful with extrapolations to other generations of Belgians (e.g., older people who actively experienced the major linguistic crises). Nevertheless, it is meaningful to keep in mind these social circumstances when proposing interventions.

#### Intergroup contact

One of the most promising ways to boost positive outgroup emotions might be through intergroup contact (Allport, 1954). As Cehajic and colleagues (2008) revealed, an increase in willingness to understand how the other party might feel (i.e., outgroup empathy) and the development of trust both play crucial mediating roles in linking intergroup contact to intergroup forgiveness. Similarly, Swart and colleagues (2011) presented longitudinal evidence that intergroup contact leads to more positive outgroup attitudes via more outgroup empathy, and Tam and colleagues (2007; 2009) indicated that (both direct and indirect) intergroup contact exerts its beneficial effects on forgiveness and approach tendencies towards the other group through more trust in and less anger towards the outgroup.

As such, contact-based interventions could endorse ‘people-to-people’ activities that bring together ordinary Flemings and Walloons to meet and collaborate on various projects that aim to consolidate reconciliation (see Gawerc, 2006). The fact that also indirect experiences with outgroup members (i.e., extended intergroup contact; Wright, Aron, McLaughlin-Volpe, & Ropp, 1997) might lead to more positive outgroup emotions and reconciliation is especially encouraging. Indeed, due to geographical segregation, there are only limited opportunities for direct contact between Flemings and Walloons. However, even knowledge of an ingroup member having a close relationship with an outgroup member can have the power to boost mutual trust and empathy, and, eventually, reconciliation.

#### The storytelling method

The storytelling method, another intervention strategy focusing on the ‘working-through’ process via meetings and lectures, has also shown to improve intergroup trust and reconciliation (Bar-On & Kassem, 2004). Storytelling serves as a transformative process and as a reflective tool. The stories are highly structured, formal ways of transmitting information and as such inform us about the world of the opposing group (i.e. enhancing perspective taking) in an atmosphere characterized by mutual trust. Moreover, because the storytelling process helps the participants to better understand the deeper, underlying issues of the conflict, it allows for the development of emotional involvement, releasing feelings of anger and hatred towards the other group. Also, the storytelling approach highlights the intergenerational aspect of the conflict and frames it within the specific macro-societal context (Bar-On & Kassem, 2004). This strategy might encourage the establishment of a constructive dialogue between the two linguistic communities in Belgium that is based on trust and empathy, and the specific focus on positive intergroup emotions and experiences might create the positive intergroup climate that is essential for truly reconciling Flemings and Walloons.

Notwithstanding the fact that our results suggest that (contact or storytelling) interventions should primarily focus on positive outgroup emotions, the reduction of outgroup anger, resentment and hate is also necessary, and might be of more relevance in violent conflict situations, such as in the former Yuguslavia (see Cehajic et al., 2008) or in the Middle East (see Halperin, 2011). In these contexts, reconciliation requires a change in the collective emotional orientations of anger, hostility, and dehumanization which often dominate societies in intractable and violent conflict (Bar-Tal, 2013; Bar-Tal et al., 2007). In Belgium, the conflict is far less dictated by animosity and resentment, and therefore we believe it is vital to develop a shared and common emotional orientation that reflects a positive outlook on future cooperative relations with the other linguistic group, rather than the mere reduction of negative outgroup emotions. Through this, social norms of tolerance, respect, dialogue, and mutual trust can develop and form the cement of our society, which has the potential to hold Flemings and Walloons together.

#### Focus on individual needs

Our findings also illustrate that, besides intergroup emotions, the psychological aspect of reconciliation is crucial, and it is the duty of social and political psychologists to shed light on the various individual-level factors that play a major function in the development of reconciliation attitudes (see also, Bar-Tal, 2000). Indeed, not everyone is equally prone to reconcile, and individual differences in cognitive style and authoritarian ideology for a large part determine our feelings, emotions, attitudes, and probably also our behaviors towards the other group. Our results indicated that especially individuals with a high intolerance for ambiguity and a high need for cognitive closure are reluctant to feel reconciled towards the outgroup, via their stronger social-cultural conservative worldview, and (albeit to a lesser extent) their higher levels of economic-hierarchical conservative worldview and their stronger belief in essence. These individuals form a challenging group to target for intervention purposes set out to improve intergroup relations between Flemings and Walloons.

## Conclusion

In conclusion, social psychology has recently made some advances towards clarifying the complex nature of reconciliation between social groups (previously) in conflict (see Nagda et al., 2006; Pittinsky et al., 2011; Tropp & Mallet, 2011). Such developments have been extremely fruitful, and our current contribution neatly builds on them. Specifically, we attempted to draw attention to the hitherto less explored but nevertheless important social psychological predictors of intergroup reconciliation attitudes. Looking into the non-violent linguistic conflict in Belgium from both sides of the conflict, we showed that, in general (i.e., for Flemings and Walloons alike), support for reconciliation stems from more positive and less negative outgroup emotions, an emotional pattern which is more prevalent among individuals with less rigid cognitive styles, as they adhere to less conservative and more progressive ideological belief systems. As such, this study aims to be the first of many examining the fundamental role of individual differences and intergroup emotions in promoting positive intergroup outcomes.

## Additional Files

The additional files for this article can be found as follows:

10.5334/pb.333.s1 Supplementary Table SEM: Overview of the Indirect and Total effects on Reconciliation.

## Footnotes

The correlation between RWA and SDO tends to be larger in countries characterized by strong, as opposed to weak, ideological contrasts (see e.g., Duriez, Van Hiel, & Kossowska, 2005).

Whereas the terms “Flemish-speaking” and “French-speaking” refer to linguistic groups, the terms “Flemings” and “Walloons” refer to the regional structuring of Belgium. We acknowledge that the French-speaking community extends beyond the limits of the Walloon geographical region to also include Brussels’ residents.

Respondents that were not born in Belgium were excluded from all analyses.

It is worth explaining why we do not report the widely popular CFI index in the subsequent analyses. On his website (http://davidakenny.net/cm/fit.htm), David Kenny has pointed out that the use of incremental fit indices such as the CFI is inappropriate whenever the RMSEA of the null model is smaller than 0.158, which is the case for all reported models. As such, reporting the CFI, popular as it may be, would lead to erroneous conclusions about the model fit.
