# Supplementary material for: Intergroup Reconciliation between Flemings and Walloons: The Predictive Value of Cognitive Style, Authoritarian Ideology, and Intergroup Emotions
Source: Psychol Belg. 2017 Nov 21;57(3):132–55. doi: 10.5334/pb.333 (PMC6194543; doi:10.5334/pb.333)
Supplement: Supplementary Table — SEM: Overview of the Indirect and Total effects on Reconciliation. [file pb-57-3-333-s1.pdf]

Supplementary Table

SEM: Overview of the Indirect and Total effects on Reconciliation

**Indirect Effect:**		**Flemish Sample (*N* = 310)**				**Walloon Sample (*N* = 329)**			
**Predictor:**	**Mediator:**	**Est.**	**SE**	**Std. Est.**	***p***	**Est.**	**SE**	**Std. Est.**	***p***
NFC	RWA	-0.019	0.023	-0.037	.405	-0.023	0.015	-0.048	.202
	ESS	-0.025	0.013	-0.049	.061	-0.011	0.009	-0.024	.041
	SDO	-0.005	0.007	-0.010	.463	-0.004	0.012	-0.008	.163
NFC	Empathy	-0.006	0.011	-0.011	.608	0.004	0.009	0.008	.650
	Trust	-0.023	0.018	-0.045	.211	-0.005	0.011	-0.011	.612
	Anger	-0.007	0.005	-0.013	.181	-0.003	0.005	-0.006	.525
RWA	Empathy	-0.022	0.011	-0.075	.041	-0.015	0.008	-0.045	.057
	Trust	-0.004	0.013	-0.015	.738	-0.011	0.009	-0.033	.211
	Anger	-0.007	0.005	-0.023	.135	-0.014	0.008	-0.040	.071
ESS	Empathy	-0.029	0.012	-0.065	.014	-0.010	0.008	-0.024	.213
	Trust	-0.034	0.019	-0.076	.073	-0.006	0.009	-0.015	.498
	Anger	-0.009	0.005	-0.021	.085	-0.006	0.005	-0.014	.221
SDO	Empathy	-0.005	0.008	-0.015	.547	-0.010	0.008	-0.028	.202
	Trust	-0.039	0.016	-0.121	.013	-0.023	0.011	-0.065	.041
	Anger	0.003	0.003	0.009	.344	0.006	0.004	0.014	.163
Total Effect:		Flemish Sample (*N* = 310)				Walloon Sample (*N* = 329)			
Predictor:		Est.	SE	Std. Est.	*p*	Est.	SE	Std. Est.	*p*
NFC		-0.023	0.036	-0.046	.518	-0.051	0.033	-0.107	.128
RWA		-0.027	0.032	-0.091	.399	-0.046	0.029	-0.134	.112
ESS		-0.141	0.044	-0.315	.001	-0.039	0.028	-0.094	.167
SDO		-0.064	0.030	-0.197	.033	-0.142	0.043	-0.406	.001

Note: NFC: need for closure; ESS: essentialism; RWA: right-wing authoritarianism; SDO: social dominance orientation
